# A Highly Sensitive and Specific Rapid Lateral Flow Assay for the HIV Integrase Strand Transfer Inhibitors Bictegravir, Cabotegravir, and Dolutegravir

**DOI:** 10.1155/arat/4809477

**Published:** 2026-10-25

**Authors:** Xierong Wei, Ae S. Youngpairoj, Richard Haaland, Chuong Dinh, Hetal K. Patel, Jose J. Perez, Kristin Brown, Thomas H. Vanderford, Jeffrey A. Johnson

**Affiliations:** ^1^ Division of HIV Prevention, Laboratory Branch CDC Atlanta Georgia USA cdc.gov; ^2^ Division of Global HIV and TB, International Laboratory Branch CDC Atlanta Georgia USA cdc.gov

## Abstract

**Background:**

Second‐generation HIV‐1 integrase strand transfer inhibitors (INSTIs)—bictegravir (BIC), cabotegravir (CAB), and dolutegravir (DTG)—are leading components of highly potent antiretroviral therapy, and CAB is also used for long‐acting chemoprevention. Rapid detection of clinically relevant INSTI concentrations could support point‐of‐care assessment and real‐time clinical decision‐making. We developed a lateral flow assay (LFA) that detects BIC, CAB, and DTG in whole blood and plasma and evaluated its performance against predefined concentration thresholds and high‐performance liquid chromatography‐tandem mass spectrometry (LC‐MS/MS).

**Methods:**

A single LFA cassette design was developed using a monoclonal antibody to distinguish BIC, CAB, and DTG concentrations below trough, at minimal‐to‐moderate trough, and at ideal concentrations. LFA results were read visually at 20 min and were compared with LC‐MS/MS–quantified INSTI concentrations for 124 clinical plasma specimens.

**Results:**

For 24 plasma samples from US participants receiving treatment involving one of the three INSTIs, the LFA interpretations matched all LC‐MS/MS results except for one DTG specimen, which was interpreted by LFA as 1.0–1.6 μg/mL but measured 2.1 μg/mL by LC‐MS/MS. Among 100 plasma remnants from routine national surveillance in Eswatini, the LFA correctly interpreted DTG concentrations in 95 specimens. Four specimens had DTG LC‐MS/MS concentrations slightly above the LFA‐determined range, differing by ≤ 0.1 μg/mL, and one specimen measured at 1.20 μg/mL by LC‐MS/MS was interpreted as ≥ 1.6 μg/mL by LFA. In total, 6 of 124 specimens (4.8%) had LFA‐interpreted INSTI concentrations that varied slightly from the LC‐MS/MS results, with no statistically significant difference in overall performance by Wilcoxon signed‐rank test (*W* = 8.00, *p* = 0.94, one‐tailed).

**Conclusions:**

The LFA design successfully provided clinically useful interpretations of BIC, CAB, and DTG concentrations with minor discrepancies in < 5% of samples. Importantly, all low‐concentration samples of < 1 μg/mL were correctly interpreted by LFA. The assay shows promise as a point‐of‐care tool for assessing clinically relevant INSTI concentrations.

## 1. Introduction

Antiretroviral formulations containing second‐generation integrase strand transfer inhibitors (INSTIs) have become the standard for HIV antiretroviral treatment (ART) due to favorable characteristics such as having a high genetic barrier to resistance, limited drug–drug interactions, excellent rates of virologic suppression, and favorable tolerability [1]. Moreover, long‐acting formulations for treatment and pre‐exposure prophylaxis (PrEP) that require less frequent dosing while offering extended protection can overcome adherence issues to maximize viral suppression and prevent virus acquisition [2]. Pharmacologic monitoring for the second‐generation INSTIs, bictegravir (BIC), cabotegravir (CAB), and dolutegravir (DTG), provides an objective measurement of antiretroviral drug concentrations with ART and PrEP use. Additionally, when experiencing HIV diagnostic test discrepancies, identification of INSTIs in blood samples provides evidence that ARVs may be suppressing HIV analyte targets used in diagnosis to levels that cause inconsistent diagnostic test results [[3] unpublished data].

Daily oral dosing of 50 mg BIC approved for HIV treatment achieves a mean plasma maximum concentration (*C*_max_) of 6.5 μg/mL (range 4.40–9.61 μg/mL), with a mean trough concentration of 2.32 μg/mL (1.44–3.76 μg/mL) [4]. Trough concentrations can vary and have been reported to decrease to 1.4 μg/mL with daily dosing; however, this reported lower boundary is 8 times greater than the BIC 95% inhibitory concentration (IC_95_) of 0.16 μg/mL [5]. CAB daily oral dosing of 30 mg used for situations when drug loading is desired achieves a median plasma concentration of 5.98 μg/mL (range 2.19–8.78 μg/mL) measured at 28 days of daily dosing [6]. Additionally, studies on daily oral DTG treatment have shown that the mean therapeutic concentration at 24 h (C_24_) is 1.32 μg/mL (range 1.18–1.47 μg/mL), and the C_48_ mean is 0.43 μg/mL (range 0.36–0.50 μg/mL) [7].

Long‐acting CAB (CAB‐LA) dosing for HIV PrEP consists of a 600‐mg intramuscular injection repeated every 8 weeks and achieves a median CAB plasma concentration of 3.79 μg/mL. The ideal CAB‐LA median trough concentration of 1.58 (1.39–1.80) μg/mL [8, 9] is slightly more than two times the protective concentration benchmark of 4 times the protein‐adjusted IC_90_ (PA‐IC_90_) or 0.66 μg/mL. Considerable intraparticipant pharmacokinetic variability has been observed with longitudinal CAB‐LA injections [10–12] and trough levels have been observed to fall to around the 4‐times PA‐IC_90_ concentration.

We developed a competitive lateral flow assay (LFA), and the “InTest‐Rapid,” designed to assess the presence of therapeutic or protective concentrations of BIC, CAB, and DTG in fresh whole blood or plasma from persons prescribed treatment or long‐acting PrEP (LA‐PrEP). Immunochromatographic LFAs operate on the principle of antigen–antibody interactions and are rapid tests that are low cost to produce and can be supplied in various healthcare settings or available over‐the‐counter [13]. For drug detection, a competitive format is necessary because the small INSTI molecules can only accommodate the binding of a single antibody. In the competitive format, antibodies bind to target molecules, in this case, INSTIs, to form a complex with the labeled monoclonal antibody (mAb) present in the LFA sample pad. As the mAb is bound by increasing concentrations of INSTI, it is less able to bind the manufactured mimetic INSTI analog present in the test lines (see below). If sufficient INSTI is present, it prevents the labeled antibody from accumulating at the test lines to form a visible band. Hence, rather than the appearance of test lines as is commonly seen with diagnostic LFAs, adequate INSTI results in the absence of test lines.

The InTest‐Rapid LFA was designed with two test lines comprised of a synthesized integrase inhibitor molecule (INI) designed from the core structure of the three target INSTIs and is striped at two concentrations. The specificity of the designed mAb to BIC, CAB, and DTG permits visual determination of clinically relevant blood concentrations for the three INSTIs. The high affinity of the mAb targets the < 1% of INSTI that remains unbound to plasma proteins and therefore alleviates the need to dissociate proteins bound to the INSTIs that is typically required to enable their detection [1, 9, 14]. Through empirical testing, the assay demonstrated the following interpretation of test‐line patterns:

• Two distinct test lines remain: INSTI concentrations, if present, are below expected trough concentrations.
• Only the upper test line is absent: INSTI is present in concentrations consistent with trough levels.
• Both test lines are absent: INSTI concentrations are at or above the target (optimal) concentration.

These visual results enable assessment of whether prescribed antiretrovirals are at adequate concentrations for treatment, and for CAB‐LA PrEP, whether drug levels have fallen below expected trough concentrations before the next scheduled injection. In a self‐test use case, the assay can inform users whether PrEP concentrations remain at the desired level; this may be especially useful given reports of declining trough concentrations in some men after more than 6 months of CAB‐LA administration [15].

## 2. Materials and Methods

### 2.1. Antibody Design, Specificity, and Sensitivity

We designed the INI INSTI analog from a common core structure of the CAB, BIC, and DTG molecules. For manufacturing the anti‐INSTI antibody, the INI molecule was conjugated to an immunogenic carrier molecule, Limulus Polyphemus Hemocyanin II, and used to immunize five Balb/c mice. After 100 days of immunization, serologic responses to INI were tested to determine the specificity of the responses for BIC, CAB, and DTG. Eighty hybridomas were generated from the mice and from which 20 supernatants had appreciable anti‐INI responses. Further screening of the supernatants for sensitivity and specificity to BIC, CAB, and DTG was performed by using a competitive format enzyme‐linked immunosorbent assay (ELISA) where known concentrations of BIC, CAB, and DTG in normal blood plasma would compete for binding the mAb diluted at 1:150 against 100 ng/mL immobilized INI substrate coated in the wells.

From this assessment, 10 clones with the greatest sensitivity were selected. The selected mAb was tested for specificity by ruling‐out cross‐reactivity to naturally occurring multicyclic compounds such as cholesterol, progesterone, testosterone, estradiol, and aldosterone. Specificity of this clone was further assessed for cross‐reactivity against the antiretroviral drugs elvitegravir, raltegravir, efavirenz, nevirapine, rilpivirine, emtricitabine, tenofovir, darunavir, etravirine, lopinavir, and ritonavir. Ultimately, one clone that provided the widest linear range of detection for all three INSTIs was selected for test development.

### 2.2. Design of the LFA Cassette

The competitive LFA to detect DTG, BIC, and CAB was designed as a cassette‐style laminated strip featuring a sample pad for blood and buffer absorption, a conjugate pad housing the colloidal gold‐labeled INSTI‐specific antibody, and a nitrocellulose membrane containing the test and control lines (see Figure 1). The test lines are the INI mimetic INSTI antigen striped at different concentrations to provide the gradated responses to different INSTI concentrations. Anti‐IgG is striped in the uppermost, third line as the test control. Briefly, the assay principle is that the sample is introduced into the sample port, followed by running buffer. When this fluid front reaches the conjugate pad, the gold nanoparticle‐labeled (nanoComposix) anti‐INI mAb is mobilized with the liquid flow. At this juncture, any INSTI in the sample will begin binding the anti‐INI mAb, thereby making the antibody unavailable to bind to the INI in the test lines. Therefore, higher INSTI concentrations result in more conjugated mAb being bound and not able to bind the test lines. The control line will bind flow‐through conjugated mAb to indicate that the antibody and sample have migrated past the test lines. Unidirectional flow is maintained by fluid absorption into the wick pad at the upper end of the cassette. The result is read 20 min after addition of the sample, prior to wick saturation and risk of fluid backflow.

**FIGURE 1:**
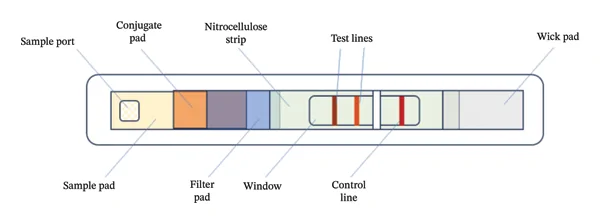
The rapid integrase strand transfer inhibitor (INSTI) lateral flow assay cassette design. Sample and buffer are added to the sample port, the conjugate pad contains the labeled anti‐INSTI monoclonal antibody, and the filter pad retains the blood cells. The sample flows to the lower and upper test lines, past the control line into the absorbing wick pad.

### 2.3. Test Materials

For point‐of‐care testing, the InTest‐Rapid procedure uses the LFA test cassette, a capillary blood collection pipette of 15 μL (for DTG) or 30 μL (for BIC/CAB), 145 μL sample dilution buffer vial (DTG), 40 μL test application pipette, and a dropper bottle of running buffer. A lancet is needed if performing a fingerstick test. For laboratory testing of venous whole blood, bench pipettes that accommodate volumes of up to 100 or 200 μL can be used. For separated plasma, a pipette that accommodates volumes up to 20 μL can be used.

### 2.4. Test Procedures

#### 2.4.1. Testing for BIC/CAB

To run the test for BIC or CAB, 30 μL of fresh fingerstick or venous whole blood is loaded to the sample port using a microsafe capillary blood pipette or laboratory pipette, respectively. For plasma testing, 18 μL is applied to the sample port using a laboratory pipette. Allow the sample to soak in the cassette for five minutes, then add five drops (∼130 μL total) chase buffer from the dropper bottle to the sample port. After 15 additional minutes, the test result is read.

#### 2.4.2. Testing for DTG

The test for DTG is similar to that of BIC/CAB except that, due to the higher affinity of the mAb to DTG, a smaller sample volume and the addition of a dilution step is required. DTG testing uses 15 μL of fresh whole blood or 9 μL of plasma, applied using a microsafe capillary pipette or a laboratory pipette, respectively. The sample is added to the dilution vial containing 145 μL of dilution buffer; after closing the vial lid, the sample is mixed well by inverting 16 times. From this diluted sample, 40 μL are added to the sample port of the cassette and incubated for five minutes. After incubation, five drops of chase buffer are added to the port, and the result is read after an additional 15 min. For higher‐sensitivity qualitative detection of DTG, the sample can be tested without dilution; however, this prevents the ability to discern if DTG is present below clinically relevant concentrations.

### 2.5. Human Subjects

We tested 24 plasma samples from persons in the United States taking BIC‐, CAB‐, or DTG‐based treatment regimens. Participants consented to provide specimens for test development in a protocol approved by the Emory Institutional Review Board (approval CR001‐IRB00009146) and with human subjects approval by the US Centers for Disease Control and Prevention (project determination #0900f3eb81dade87). Additionally, 100 plasma samples were obtained from a nationally representative household survey, Eswatini (formerly Swaziland) HIV Impact Measurement Survey (SHIMS‐3). These samples were conveniently selected from a subset of specimens sent for drug resistance genotyping. The Population‐Based HIV Impact Assessment (PHIA) survey was approved by the Institutional Review Boards at Columbia University Irving Medical Center, Westat, Inc, and the Eswatini Health and Human Research Review Board (Eswatini project approval 7263).

This project was reviewed in accordance with CDC human research protection procedures and was determined to be nonresearch (project determination #0900f3eb81aa2699). Participants were under the standard of care tenofovir–lamivudine–dolutegravir (TLD) treatment and provided informed consent for participating in the survey and for future use of their leftover specimens. INSTI concentrations for all participants were separately determined by high‐performance liquid chromatography–tandem mass spectrometry (LC‐MS/MS); however, results were not made available until LFA testing was completed.

## 3. Results

### 3.1. Determining Anti‐INI Antibody Limit of Detection

Of 10 clones which were reactive to the INI mimetic INSTI compound, one that demonstrated similar high sensitivity and slope linearity to all three INSTIs, mAb Clone 24 (3H9E4), was selected for additional testing (Figure 2). Competition testing of mAb 24 for BIC, CAB, and DTG against binding to immobilized INI antigen‐coated plates in ELISA‐based screening demonstrated desired concentration‐dependent binding to each inhibitor. The linear ranges of detection extended for 4‐5 log_10_ dilutions of the INSTIs. For BIC and CAB serial dilutions, the linear range for mAb 24 binding was 500000 pg/mL–50 pg/mL, with *R* ^2^ = 0.938 and 0.961, respectively. The linear range for DTG was 50000 pg/mL–5 pg/mL, *R* ^2^ = 0.965 (Supporting Figure S1). The lowest limit of INSTI detection for purified mAb 24 that was reproducibly discernible from wells with no drug was 50 pg/mL for BIC and CAB and 5 pg/mL for DTG.

**FIGURE 2:**
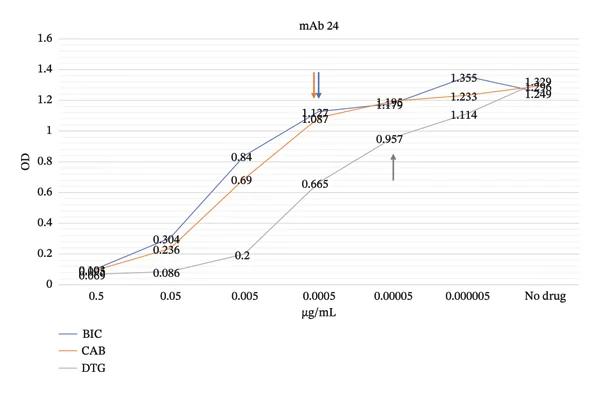
Optical density (OD) readings for concentration ranges of bictegravir, cabotegravir, and dolutegravir binding by Monoclonal Antibody 24 (mAb 24) detected in competitive ELISA. Arrows indicate the lowest concentration of each INSTI that can still be distinguished by a decline of 0.200 OD from the no‐drug background. These concentrations were selected as the limits of quantitation for each inhibitor; however, the clinically relevant concentrations are well above these lower detection limits. The order of magnitude greater sensitivity for DTG detection required sample dilution for use in the single‐design lateral flow cassette. Abbreviations: BIC, bictegravir; CAB, cabotegravir; DTG, dolutegravir; INSTI, integrase strand transfer inhibitor; ELISA, enzyme‐linked immunosorbent assay.

## 4. Specificity Testing for Anti‐INI mAb

The results of the mAb 24 ELISA testing against nontarget compounds as compared to its binding to BIC are shown in Supporting Figure S2. In this representation, each compound was individually diluted in normal human plasma at a concentration of 1.75 μg/mL and the reactivity of the antibody against each analyte, as measured by reduction in optical density (OD), was assessed using the ratio of OD without analyte/OD with analyte. This measured mAb binding by assessing whether the compounds prevent the antibody from binding the assay INI compound immobilized on the plate. Increases in this ratio indicate increased mAb 24 binding to the compound, whereas a ratio of one indicates no binding by the mAb. All nontarget compounds resulted in ratios < 1.5.

## 5. Establishing LFA Line Interpretations

We experimented with sample and buffer volumes and incubation times to obtain the desired visual loss of test lines across INSTI concentrations of 0.0–2.0 μg/mL. Our goals were for the upper test line to disappear at the low end of typical INSTI trough concentrations (0.8–1.0 μg/mL) and for both the upper and lower test line to disappear at or above 1.3 μg/mL. This pattern allowed for differentiation of low, intermediate, and high drug exposure: The presence of both test lines indicates INSTI concentrations below expected trough levels. Therefore, loss of both lines indicates concentrations consistent with high ART adherence or ideal LA‐PrEP concentrations. For CAB‐LA specifically, retention of both test lines would be interpreted as CAB near or below the 4 times PA‐IC_90_ benchmark.

Multiple concentrations of INI antigen were striped for the upper and lower test lines to acquire the differential test line sensitivities mentioned above. Ultimately, through repeated empirical testing of quantitated INSTI dilutions in whole blood, we determined that the INI antigen striped at a higher amount of 16 ng for the bottom test line and at 9 ng for the upper test line provided test responses compatible with our target performance.

We first evaluated test line performance with both whole blood and plasma spiked with known concentrations (0.0, 0.5, 0.75, 1.0, and 1.3 μg/mL) of BIC, CAB, or DTG to encompass the clinically relevant boundaries for expected target concentrations, represented by BIC testing in Supporting Figure S3. Use of fresh whole blood—no more than 2 days after collection—is important to avoid high background caused by hemoglobin release from lysed red blood cells. Initially, the test line interpretations were established to be the same for all three INSTIs with two visible lines indicating INSTI concentrations of < 1.0 μg/mL and no test line indicating concentrations ≥ 1.3 μg/mL. After testing an additional 100 clinical specimens for which INSTI concentrations were quantitated by LC‐MS/MS (not shown), the test line interpretations were adjusted slightly as described below.

The final test line pattern interpretations are described as follows and are illustrated in Figure 3. For BIC and CAB testing, the presence of two distinct test lines indicates that these INSTIs are either absent or at a concentration of < 0.8 μg/mL. One distinct lower test line is interpreted as the BIC/CAB concentration is ≥ 0.8 μg/mL to < 1.3 μg/mL, slightly to moderately above the minimally desired concentration. No distinct test line is interpreted as BIC/CAB is present at concentrations of ≥ 1.3 μg/mL.

**FIGURE 3:**
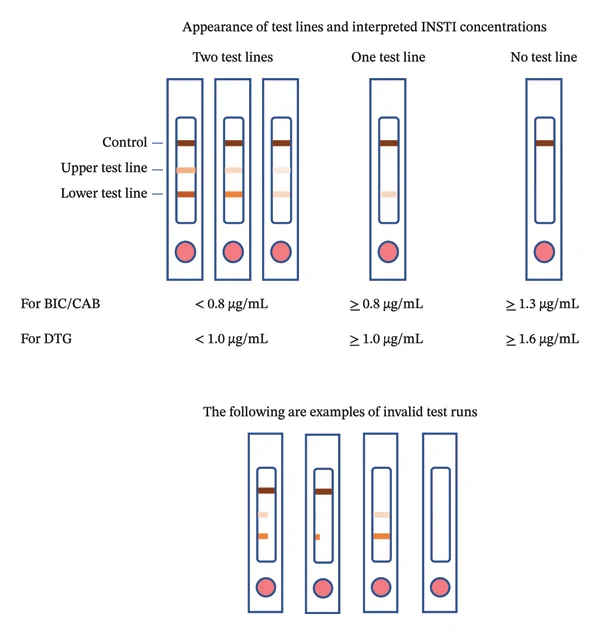
Lateral flow assay interpretations established for clinically relevant concentrations of the three INSTIs. Broken test lines or the absence of the control line invalidates the test run. Abbreviations: INSTI, integrase strand transfer inhibitor; BIC, bictegravir; CAB, cabotegravir; DTG, dolutegravir.

For DTG, the interpretations are similar except the presence of one or two test lines discerns concentrations of ≥ 1.0 to < 1.6 μg/mL or < 1.0 μg/mL, respectively. No visible test line is interpreted as DTG concentrations ≥ 1.6 μg/mL. For all testing, if no control line is present or if any line is not intact, the test run is invalid.

## 6. LFA Performance With Blinded Testing of Clinical Samples

Among the 124 clinical samples that were tested blind to LC‐MS/MS‐measured concentrations, the LFA interpretations for 118 (95.2%) samples were consistent with the range of the LC‐MS/MS results. The testing of US samples is shown in Table 1, and the Eswatini sample results are depicted in Figure 4 and Supporting Table S1. Five samples, comprising one from the US and four from Eswatini, tested slightly below but within 0.1 μg/mL of the concentrations determined by LC‐MS/MS. One Eswatini sample resulted in a higher LFA interpretation than what was quantitated by LC‐MS/MS, ≥ 1.6 vs. 1.2 μg/mL, respectively. In all, 4.8% of specimens resulted in slightly different interpretations than the LC‐MS/MS data. However, in comparing the results of the LFA concentration range interpretations relative to the INSTI concentrations quantitated by LC‐MS/MS, the overall performance of the LFA was not statistically different from the LC‐MS/MS data (Wilcoxon rank sum, *W* = 8.00, *p* = 0.94, one‐tailed), and for all discrepant sample results both tests indicated that the INSTI concentrations reflected adherent dosing. None of the samples with INSTI concentrations below expected trough as measured by LC‐MS/MS were incorrectly interpreted by the LFA as having higher concentrations.

**TABLE 1:** Lateral flow assay (LFA) test results with plasma from persons provided various INSTI‐based HIV treatment regimens.

Sample ID	INSTI used	LC‐MS/MS data (μg/mL)	BIC (μg/mL)	CAB (μg/mL)	DTG (μg/mL)
21‐C‐24‐1	BIC	0.14	< 0.8		
21‐C‐46‐1	BIC	1.6	≥ 1.3		
21‐C‐23‐1	BIC	1.9	≥ 1.3		
21‐C‐36‐1	BIC	1.9	≥ 1.3		
21‐C‐57‐1	BIC	1.9	≥ 1.3		
21‐C‐15	BIC	2.3	≥ 1.3		
21‐C‐27	BIC	2.4	≥ 1.3		
21‐C‐06‐1	BIC	3.0	≥ 1.3		
21‐C‐22‐1	BIC	4.5	≥ 1.3		
21‐C‐04‐1	BIC	4.8	≥ 1.3		
21‐C‐14‐1	BIC	5.1	≥ 1.3		
21‐C‐26‐1	BIC	5.4	≥ 1.3		
21‐C‐50	BIC	5.6	≥ 1.3		
21‐C‐07‐1	BIC	6.3	≥ 1.3		
21‐C‐30	BIC	6.4	≥ 1.3		
21‐D‐49‐1	CAB	1.8		≥ 1.3	
21‐D‐49‐2	CAB	2.1		≥ 1.3	
21‐D‐49‐3	CAB	2.5		≥ 1.3	
21‐C‐13‐1	DTG	0.81			< 1.0
21‐C‐11‐1	DTG	2.1			≥ 1.6
21‐C‐31	DTG	2.1			≥ 1.0^∗^
21‐C‐40	DTG	2.1			≥ 1.6
21‐C‐08‐1	DTG	2.5			≥ 1.6
21‐C‐12‐1	DTG	7.2			≥ 1.6

*Note:* The INSTI used in each treatment regimen is listed. The values provided in the BIC, CAB, and DTG columns are the drug concentration interpretations from the LFA test line patterns. LC‐MS/MS concentrations for each specimen are indicated but were not provided at the time of LFA testing.

Abbreviations: BIC, bictegravir; CAB, cabotegravir; DTG, dolutegravir; INSTI, integrase strand transfer inhibitor; LC‐MS/MS, high‐performance liquid chromatography–tandem mass spectrometry.

^∗^The LC‐MS/MS value is greater than 1.0 μg/mL; however, the LFA ≥ 1.0 μg/mL is interpreted as concentrations between 1.0 and < 1.6 μg/mL. Subsequent LFA testing of this individual consistently resulted in concentrations lower than that determined by LC‐MS/MS.

**FIGURE 4:**
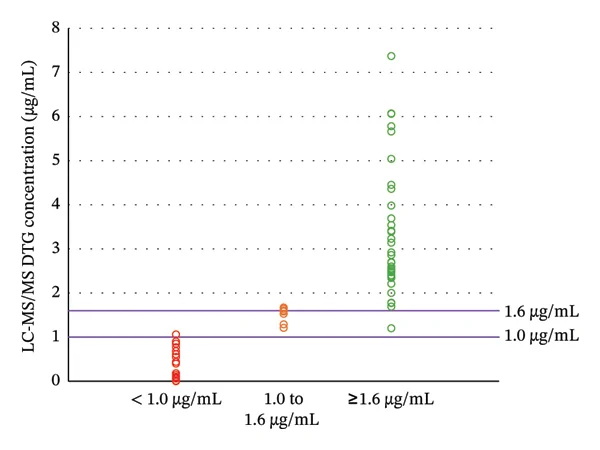
Dot plot of the LFA results compared to quantitated DTG concentrations by LC‐MS/MS testing of the Eswatini plasma. The three interpretation ranges with LFA testing: < 1.0, 1.0 to < 1.6, and ≥ 1.6 μg/mL are shown. All LFA interpretations except for one sample at > 1 μg/mL (the lowest green circle) are within a difference of 0.1 μg/mL of the quantitated LC‐MS/MS result. All samples below expected trough levels as measured by LC‐MS/MS (red circles) were correctly interpreted by the LFA. Abbreviations: LC‐MS/MS, high‐performance liquid chromatography–tandem mass spectrometry; LFA, lateral flow assay; DTG, dolutegravir.

## 7. Discussion

Using a mAb created in house, we developed a sensitive, rapid LFA to detect clinically relevant BIC, CAB, and DTG concentrations in fresh whole blood and plasma from individuals who are administered INSTI‐based ART or CAB‐LA PrEP. Antibody Clone 24 had picogram per milliliter sensitivity for the INSTIs, which permitted its direct use in blood with no need to manipulate the sample to dissociate protein bound to the INSTIs. Because INSTIs are highly bound to plasma proteins, the high sensitivity represents a significant feature that allowed the design of a simple lateral flow format test for use in settings where sample processing is not available. Sensitivity testing revealed that this mAb was more sensitive to DTG than to BIC and CAB; therefore, we established that for a single cassette design, a dilution step was needed for DTG testing.

The good linear trends of mAb 24 binding to the three INSTIs (all *R* ^2^ > 0.93), as well as nearly parallel slopes, made it possible to create a single test strip design suitable for all three inhibitors. By titering the concentrations of the INI antigen in the test lines, we were able to obtain an acceptable test response over clinically relevant INSTI thresholds. A two test‐line system was created to provide additional assurance around relevant clinical concentrations that a single test line would not provide due to pharmacokinetic variability. This was observed with the more critical concentrations near the bottom of trough levels in which INSTI concentrations below this cutoff were correctly interpreted by both lines remaining present. Additionally, the infrequent occurrence of a single test line being observed when drug concentrations were above trough levels underestimated those INSTI concentrations; however, the INSTIs were interpreted as within expected trough and would not have been a concern for clinical decision‐making. For testing CAB‐LA PrEP, observing a change from no lines present to the appearance of one and two lines may forecast that CAB pharmacokinetics is trending downward over time. While an intermediate/trough concentration range is provided with the LFA, the healthcare provider can make the determination if distinguishing INSTI concentrations in this range is of particular value to them.

We found that among the 124 clinical samples evaluated, five specimen concentrations interpreted by LFA were slightly lower than their quantitation by LC‐MS/MS, but all were within expected trough levels for the INSTIs. For one other sample, the LFA scored a higher DTG concentration than what was measured by LC‐MS/MS; however, here too, the INSTI concentration was above the expected trough level (> 1 μg/mL). Therefore, the LFA discrepancies did not result in a concerning interpretation in which inadequate ARV concentrations were not identified. On the contrary, all samples with undetectable or low INSTI concentrations by LC‐MS/MS were correctly interpreted by the LFA as being less than expected trough concentrations.

From our vantage point of HIV testing within public health systems, multiple use cases exist for a rapid point‐of‐care antiretroviral drug test. For one, in cases with ambiguous HIV diagnostic testing, a quick assessment by the clinical diagnostic laboratory can determine if ARVs are present that may be interfering with the expression of HIV infection targets used in diagnosis. In clinical trial participant testing, assessing adherence to study treatment or PrEP to ensure efficacy outcomes can be interpreted correctly. Furthermore, the rapid test can be applied for surveillance uses as a metric of treatment adherence prevalence. The LFA test also has potential as a self‐test to ensure users that protective levels are maintained between LA‐PrEP visits. Additionally, the rapid assessment of ARV levels in persons prescribed treatment who are experiencing viremia could help prioritize those individuals for genotyping as they are most likely to develop drug resistance (see Supporting Figure S4). Being able to make a decision at clinic visits of whether a change in regimen is warranted in the absence of genotyping may help to reduce proliferation of drug resistance.

The data presented here have limitations. While the assay was developed with INSTI‐spiked whole blood and plasma, the clinical testing presented was largely performed on plasma. Clinical testing using fresh whole blood is underway and will be reported elsewhere. We found that some persons consistently tested lower with the LFA than what LC‐MS/MS quantitated. The differences were small, but it is not understood what factors may result in the rapid test interpreting lower INSTI detection levels for some individuals. All discrepancies were seen with DTG testing and, therefore, the dilution step may have introduced error if the sample is not diluted properly. Conversely, there is up to 15% error in LC‐MS/MS accuracy, and such variation may impact quantitation of concentrations that border the LFA interpretation thresholds. Finally, the LFA interpretations in this evaluation were made by testers who are experienced in reading rapid tests. We anticipate that in the absence of experience with competitive tests, a learning period will be needed to properly interpret the competitive assay for which the disappearance of lines is in contrast to the reactive appearance of lines in conventional diagnostic rapid tests.

In conclusion, the INSTI rapid LFA (InTest‐Rapid) performance was acceptable in interpreting clinically significant concentrations of the INSTIs BIC, CAB, and DTG in plasma samples. The slight differences in ∼5% of test results did not lead to troubling discrepancies between the LFA and gold‐standard LC‐MS/MS concentrations and had no significant impact on overall test performance. Establishing LFA test parameters for use with both whole blood and plasma allows for flexible application in various settings. The ability to quickly obtain ARV concentrations for real‐time clinical management of treatment and biomedical prevention may help providers optimize patient care. Furthermore, easily obtaining biologic evidence of ARV use in clinical studies and in investigating diagnostic dilemmas can help avoid clinical interpretations that are based on the findings that have been impacted by unrecognized ARV use. Incorporating objective biologic evidence of ARV use into study protocols and clinical evaluations would improve diagnostic accuracy and study findings.

## Author Contributions

Xierong Wei: development, performance testing, and verifying final reagent volumes and test parameters for the LFA, and LFA testing of the US clinical samples; Ae S. Youngpairoj: screening and selection of antibody clones, establishing the ELISA for determining antibody sensitivity and specificity, determining the antibody limits of quantitation, performing the interfering substances testing, and assisting with verifying LFA test parameters; Richard Haaland, Chuong Dinh, and Jose J. Perez: performing and reviewing the LC‐MS/MS testing for INSTI concentrations; Hetal K. Patel: oversight of the Eswatini sample testing and approval of all laboratory data for SHIMs‐3; Jose J. Perez: LFA testing of the Eswatini samples; Thomas H. Vanderford: LFA and ELISA test management and data review and input on lateral flow design; Jeffrey A. Johnson: concept of the INSTI LFA format, design of the INI antigen, overall validation procedure for antibody evaluation, review and approval of antibody selection and performance parameters, and principal manuscript author.

## Funding

This work was funded by the Centers for Disease Control and Prevention, intramural funding.

## Disclosure

The findings and conclusions in this article are those of the authors and do not necessarily represent the official views of the US Centers for Disease Control and Prevention and the US Department of Health and Human Services.

## Conflicts of Interest

Jeffrey A. Johnson, Xierong Wei, and Ae S. Youngpairoj are inventors of the monoclonal antibody used in the development of the INSTI lateral flow assay. The other authors declare no conflicts of interest.

## Supporting Information

Additional supporting information can be found online in the Supporting Information section.

## Supporting information


**Supporting Information** Figure S1: Monoclonal Antibody 24 (mAb 24) reactivity against linear dilutions of BIC, CAB, and DTG as measured by competitive ELISA. Figure S2: The absence of Monoclonal Antibody 24 (mAb 24) reactivity to other antiretroviral compounds and naturally occurring polycyclic compounds. Figure S3: Lateral flow test line appearance with bictegravir (BIC) dilutions in human whole blood and plasma. Table S1: Line listing of LC‐MS/MS data as compared to the LFA interpretations for 100 plasma specimens from persons prescribed dolutegravir (DTG)‐based antiretroviral therapy (tenofovir–lamivudine–dolutegravir). Figure S4: Flow diagram of viral loads relative to LFA interpretations for 100 plasma specimens from persons prescribed dolutegravir (DTG)‐based antiretroviral therapy depicting the minor fraction of individuals with both high viral load and DTG concentrations.

## Data Availability

The data that support the findings of this study are available on request from the corresponding author. The data are not publicly available due to privacy or ethical restrictions.
