# Supplementary material for: A Highly Sensitive and Specific Rapid Lateral Flow Assay for the HIV Integrase Strand Transfer Inhibitors Bictegravir, Cabotegravir, and Dolutegravir
Source: AIDS Res Treat. 2026 Oct 25;2026:4809477. doi: 10.1155/arat/4809477 (PMC13695850; doi:10.1155/arat/4809477)
Supplement: Supplementary file 1 [file ARAT-2026-4809477-s001.zip › Figure S1.pdf]

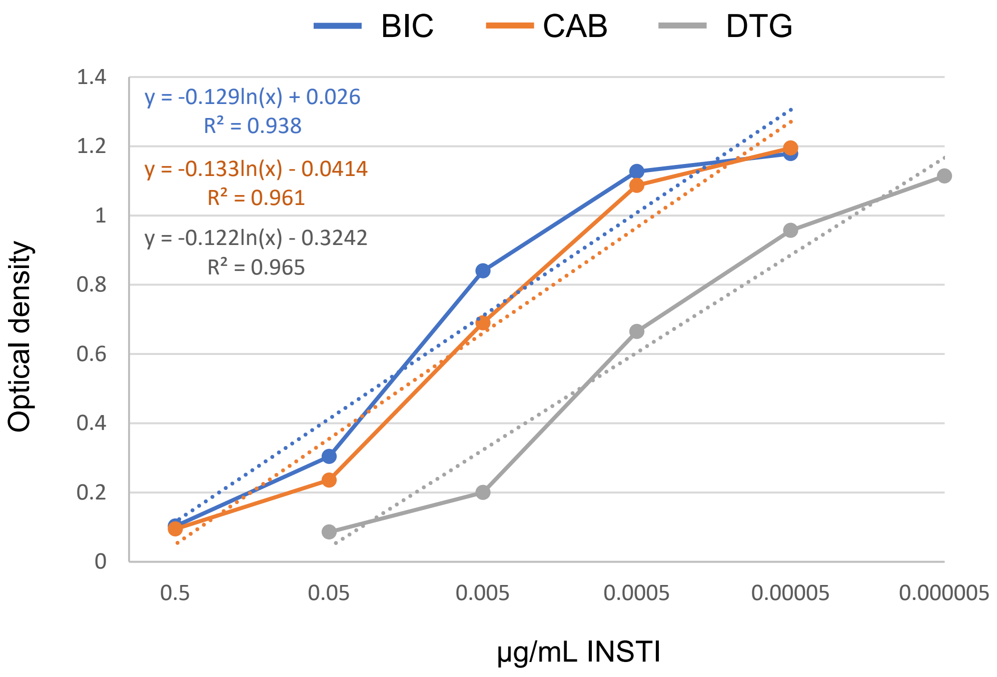
BIC
CAB
DTG
y = -0.129ln(x) + 0.026
R² = 0.938
y = -0.133ln(x) - 0.0414
R² = 0.961
y = -0.122ln(x) - 0.3242
R² = 0.965
1.4
1.2
1
0.8
0.6
0.4
0.2
0
Optical density
0.5
0.05
0.005
0.0005
0.00005
0.000005
µg/mL INSTI
