# Supplementary material for: A Highly Sensitive and Specific Rapid Lateral Flow Assay for the HIV Integrase Strand Transfer Inhibitors Bictegravir, Cabotegravir, and Dolutegravir
Source: AIDS Res Treat. 2026 Oct 25;2026:4809477. doi: 10.1155/arat/4809477 (PMC13695850; doi:10.1155/arat/4809477)
Supplement: Supplementary file 1 [file ARAT-2026-4809477-s001.zip › Figure S2.pdf]

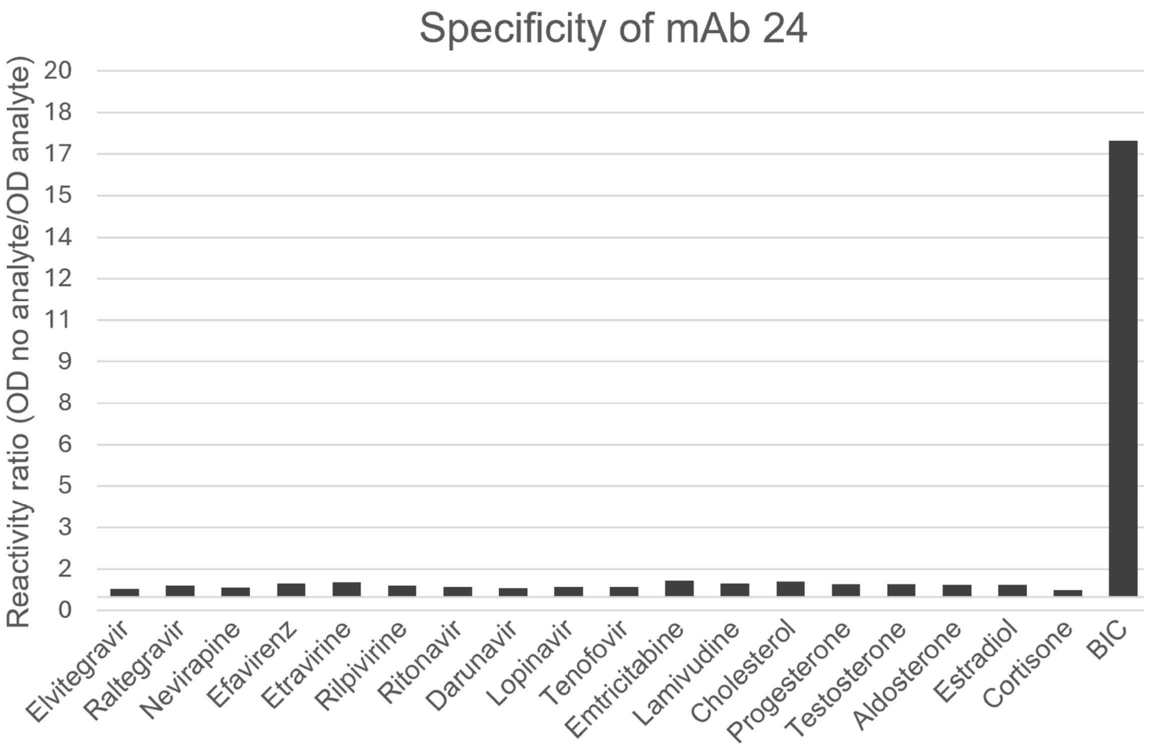

Specificity of mAb 24
Reactivity ratio (OD no analyte/OD analyte)
20
18
17
15
14
12
11
9
8
6
5
3
2
0
Elvitegravir
Raltegravir
Nevirapine
Efavirenz
Etravirine
Rilpivirine
Ritonavir
Darunavir
Lopinavir
Tenofovir
Emtricitabine
Lamivudine
Cholesterol
Progesterone
Testosterone
Aldosterone
Estradiol
Cortisone
BIC
