# Supplementary material for: A Highly Sensitive and Specific Rapid Lateral Flow Assay for the HIV Integrase Strand Transfer Inhibitors Bictegravir, Cabotegravir, and Dolutegravir
Source: AIDS Res Treat. 2026 Oct 25;2026:4809477. doi: 10.1155/arat/4809477 (PMC13695850; doi:10.1155/arat/4809477)
Supplement: Supplementary file 1 [file ARAT-2026-4809477-s001.zip › Figure S3.pdf]

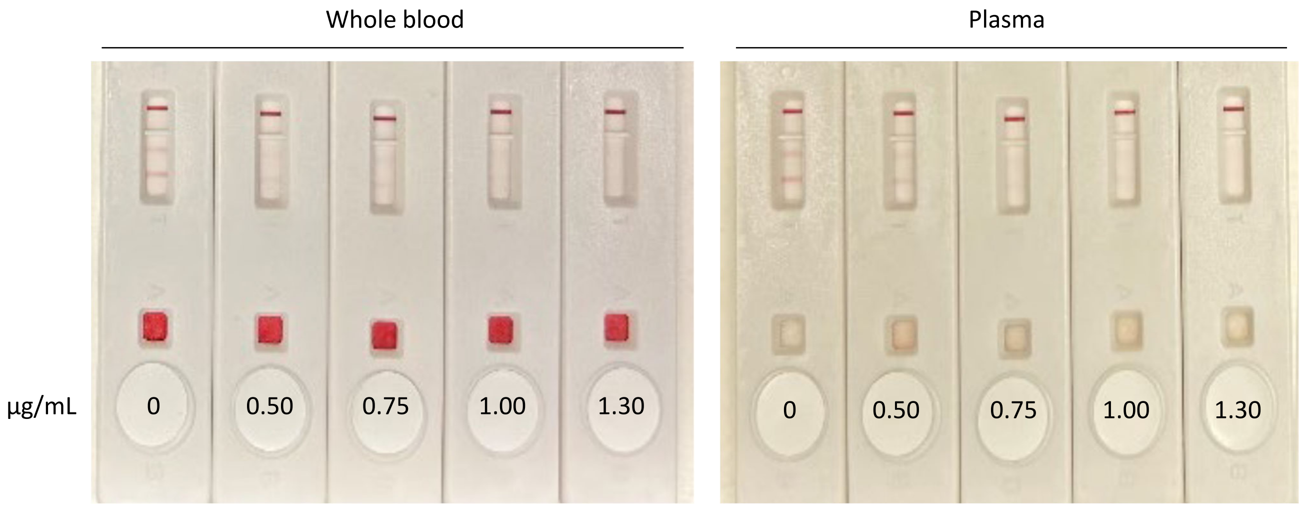
Whole blood
Plasma
µg/mL
0
0.50
0.75
1.00
1.30
0
0.50
0.75
1.00
1.30
