# Supplementary material for: A Highly Sensitive and Specific Rapid Lateral Flow Assay for the HIV Integrase Strand Transfer Inhibitors Bictegravir, Cabotegravir, and Dolutegravir
Source: AIDS Res Treat. 2026 Oct 25;2026:4809477. doi: 10.1155/arat/4809477 (PMC13695850; doi:10.1155/arat/4809477)
Supplement: Supplementary file 1 [file ARAT-2026-4809477-s001.zip › Figure S4.pdf]

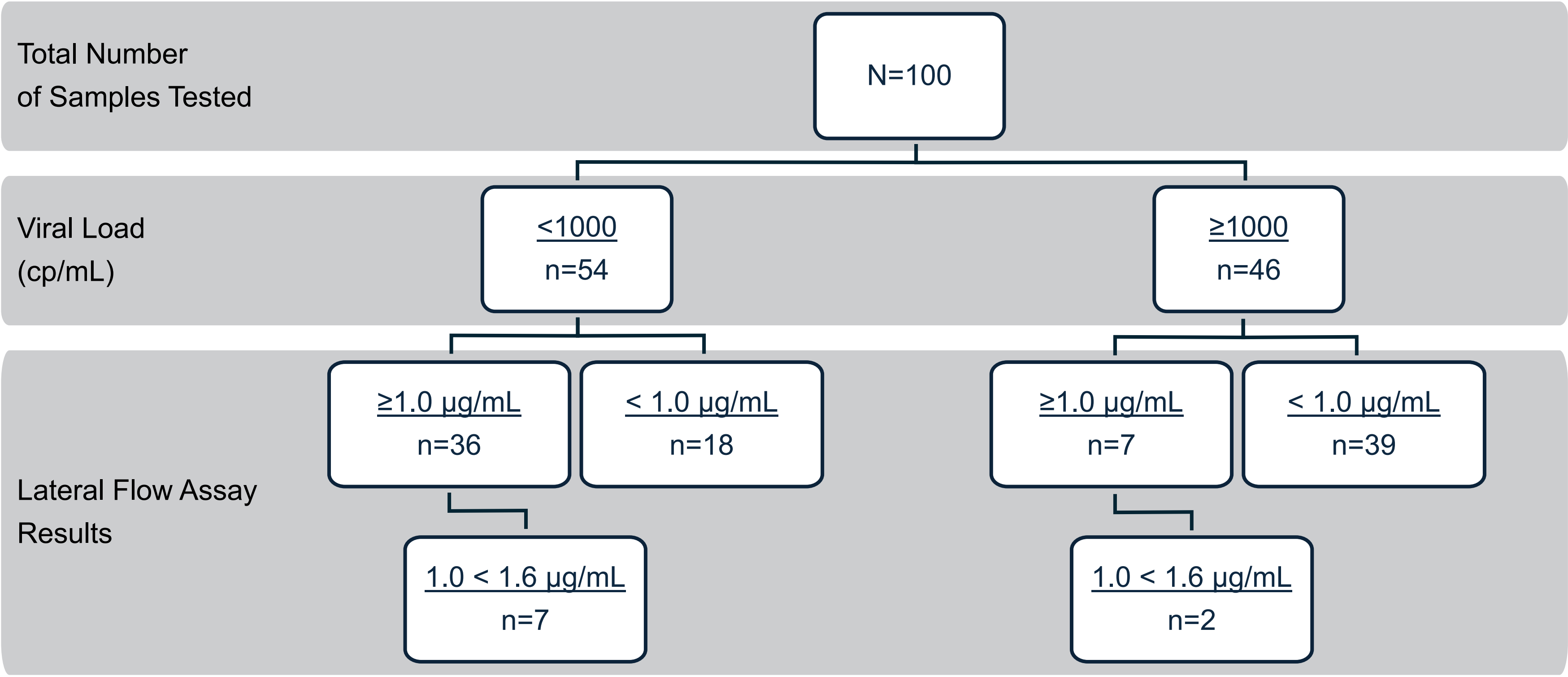

Total Number
of Samples Tested
N=100
Viral Load
(cp/mL)
<1000
n=54
≥1000
n=46
Lateral Flow Assay
Results
≥1.0 µg/mL
n=36
< 1.0 µg/mL
n=18
≥1.0 µg/mL
n=7
< 1.0 µg/mL
n=39
1.0 < 1.6 µg/mL
n=7
1.0 < 1.6 µg/mL
n=2
