# Supplementary material for: A Highly Sensitive and Specific Rapid Lateral Flow Assay for the HIV Integrase Strand Transfer Inhibitors Bictegravir, Cabotegravir, and Dolutegravir
Source: AIDS Res Treat. 2026 Oct 25;2026:4809477. doi: 10.1155/arat/4809477 (PMC13695850; doi:10.1155/arat/4809477)
Supplement: Supplementary file 1 [file ARAT-2026-4809477-s001.zip › LFA for INSTIs_supplemental_JJohnson.docx]

Supplemental information

Figure S1. The linear ranges of mAb 24 binding to BIC (blue), CAB (orange), and DTG (gray). All correlation coefficients have R^2^ values above 0.93 and the slopes are nearly parallel which permitted a single test cassette design for all three INSTIs. The quantitative ranges extend ~3log_10_ lower than clinically relevant protein adjusted INSTI concentrations in plasma [13]. Abbreviations: BIC, bictegravir; CAB, cabotegravir; DTG, dolutegravir; INSTI, integrase strand transfer inhibitor; mAb, monoclonal antibody.

Specificity of mAb 24

Reactivity ratio (OD no analyte/OD analyte)

|  | No drug/Drug |
| --- | --- |
| Elvitegravir | 0.803982 |
| Raltegravir | 0.903772 |
| Nevirapine | 0.835198 |
| Efavirenz | 0.98 |
| Etravirine | 1.02224 |
| Rilpivirine | 0.915595 |
| Ritonavir | 0.856345 |
| Darunavir | 0.82923 |
| Lopinavir | 0.853434 |
| Tenofovir | 0.865922 |
| Emtricitabine | 1.0842 |
| Lamivudine | 0.974265 |
| Cholesterol | 1.04774 |
| Progesterone | 0.962519 |
| Testosterone | 0.948594 |
| Aldosterone | 0.938743 |
| Estradiol | 0.93704 |
| Cortisone | 0.756778 |
| BIC | 16.9853 |

Figure S2. The specificity of mAb 24 was assessed by competitive enzyme immunoassay against other INSTIs (elvitegravir, raltegravir), other antiretroviral drug classes, and naturally occurring polycyclic compounds individually dissolved in human blood plasma at 1.75 µg/mL. The reactivity to these compounds is represented as a ratio of the reduction in OD measurements against plasma alone over reactivity to compound in plasma (competitive inhibition). A ratio of 1 depicts no compound binding that interferes with the mAb binding to INI antigen immobilized on the plates. The reactivity to bictegravir (BIC) is shown for comparison. Abbreviations: mAb, monoclonal antibody; OD, optical density; INSTI, integrase strand transfer inhibitor; INI, integrase inhibitor.

Figure S3. The test line patterns observed in whole blood and plasma represented by testing bictegravir (BIC) spiked at the drug concentrations indicated at the bottom. The monoclonal antibody has the lowest affinity for BIC and therefore it was the most difficult integrase strand transfer inhibitor to establish the desired test line patterns across this therapeutic range. Whole blood was applied at 30 µL/test and plasma was applied at 18 µL/test to account for the difference in blood cell volume.

Table S1. Comparison of lateral flow assay (LFA) results and LC-MS/MS concentrations determined from testing plasma from 100 persons prescribed DTG-based treatment. The values provided in the LFA DTG column are the drug concentration interpretations from the LFA banding patterns. LC-MS/MS concentrations determined for each participant are indicated; however, results were not revealed until LFA results were reported.

| **Sample #** | **LC-MS/MS**  **DTG (µg/mL)** | **LFA DTG Result** |
| --- | --- | --- |
| 12 | 0.00 | <1.0 µg/mL |
| 13 | 0.00 | <1.0 µg/mL |
| 14 | 0.00 | <1.0 µg/mL |
| 17 | 0.00 | <1.0 µg/mL |
| 18 | 0.00 | <1.0 µg/mL |
| 21 | 0.00 | <1.0 µg/mL |
| 22 | 0.00 | <1.0 µg/mL |
| 23 | 0.00 | <1.0 µg/mL |
| 24 | 0.00 | <1.0 µg/mL |
| 27 | 0.00 | <1.0 µg/mL |
| 30 | 0.00 | <1.0 µg/mL |
| 38 | 0.00 | <1.0 µg/mL |
| 42 | 0.00 | <1.0 µg/mL |
| 43 | 0.00 | <1.0 µg/mL |
| 47 | 0.00 | <1.0 µg/mL |
| 49 | 0.00 | <1.0 µg/mL |
| 52 | 0.00 | <1.0 µg/mL |
| 53 | 0.00 | <1.0 µg/mL |
| 55 | 0.00 | <1.0 µg/mL |
| 56 | 0.00 | <1.0 µg/mL |
| 57 | 0.00 | <1.0 µg/mL |
| 63 | 0.00 | <1.0 µg/mL |
| 67 | 0.00 | <1.0 µg/mL |
| 68 | 0.00 | <1.0 µg/mL |
| 70 | 0.00 | <1.0 µg/mL |
| 73 | 0.00 | <1.0 µg/mL |
| 74 | 0.00 | <1.0 µg/mL |
| 81 | 0.00 | <1.0 µg/mL |
| 96 | 0.00 | <1.0 µg/mL |
| 99 | 0.00 | <1.0 µg/mL |
| 4 | 0.01 | <1.0 µg/mL |
| 8 | 0.01 | <1.0 µg/mL |
| 16 | 0.01 | <1.0 µg/mL |
| 20 | 0.01 | <1.0 µg/mL |
| 29 | 0.01 | <1.0 µg/mL |
| 33 | 0.01 | <1.0 µg/mL |
| 36 | 0.01 | <1.0 µg/mL |
| 45 | 0.01 | <1.0 µg/mL |
| 77 | 0.01 | <1.0 µg/mL |
| 88 | 0.01 | <1.0 µg/mL |
| 90 | 0.01 | <1.0 µg/mL |
| 92 | 0.01 | <1.0 µg/mL |
| 75 | 0.06 | <1.0 µg/mL |
| 100 | 0.06 | <1.0 µg/mL |
| 62 | 0.09 | <1.0 µg/mL |
| 94 | 0.10 | <1.0 µg/mL |
| 1 | 0.14 | <1.0 µg/mL |
| 51 | 0.18 | <1.0 µg/mL |
| 72 | 0.40 | <1.0 µg/mL |
| 66 | 0.44 | <1.0 µg/mL |
| 48 | 0.55 | <1.0 µg/mL |
| 41 | 0.61 | <1.0 µg/mL |
| 54 | 0.68 | <1.0 µg/mL |
| 59 | 0.77 | <1.0 µg/mL |
| 85 | 0.85 | <1.0 µg/mL |
| 79 | 0.90 | <1.0 µg/mL |
| 46 | 1.06 | <1.0 µg/mL |
| 82 | 1.20 | ≥1.6 µg/mL |
| 39 | 1.21 | ≥1.0 µg/mL |
| 71 | 1.29 | ≥1.0 µg/mL |
| 98 | 1.53 | ≥1.0 µg/mL |
| 40 | 1.58 | ≥1.0 µg/mL |
| 2 | 1.60 | ≥1.0 µg/mL* |
| 37 | 1.64 | ≥1.0 µg/mL* |
| 80 | 1.67 | ≥1.0 µg/mL* |
| 69 | 1.69 | ≥1.6 µg/mL |
| 95 | 1.69 | ≥1.6 µg/mL |
| 28 | 1.77 | ≥1.6 µg/mL |
| 26 | 1.78 | ≥1.6 µg/mL |
| 84 | 2.00 | ≥1.6 µg/mL |
| 60 | 2.21 | ≥1.6 µg/mL |
| 11 | 2.34 | ≥1.6 µg/mL |
| 50 | 2.37 | ≥1.6 µg/mL |
| 65 | 2.40 | ≥1.6 µg/mL |
| 64 | 2.47 | ≥1.6 µg/mL |
| 35 | 2.50 | ≥1.6 µg/mL |
| 93 | 2.53 | ≥1.6 µg/mL |
| 34 | 2.56 | ≥1.6 µg/mL |
| 9 | 2.60 | ≥1.6 µg/mL |
| 15 | 2.60 | ≥1.6 µg/mL |
| 10 | 2.69 | ≥1.6 µg/mL |
| 19 | 2.70 | ≥1.6 µg/mL |
| 86 | 2.70 | ≥1.6 µg/mL |
| 89 | 2.86 | ≥1.6 µg/mL |
| 78 | 2.92 | ≥1.6 µg/mL |
| 44 | 3.14 | ≥1.6 µg/mL |
| 7 | 3.23 | ≥1.6 µg/mL |
| 32 | 3.39 | ≥1.6 µg/mL |
| 25 | 3.41 | ≥1.6 µg/mL |
| 97 | 3.54 | ≥1.6 µg/mL |
| 91 | 3.69 | ≥1.6 µg/mL |
| 31 | 3.98 | ≥1.6 µg/mL |
| 58 | 4.36 | ≥1.6 µg/mL |
| 83 | 4.45 | ≥1.6 µg/mL |
| 87 | 5.04 | ≥1.6 µg/mL |
| 3 | 5.66 | ≥1.6 µg/mL |
| 61 | 5.78 | ≥1.6 µg/mL |
| 5 | 6.06 | ≥1.6 µg/mL |
| 76 | 6.07 | ≥1.6 µg/mL |
| 6 | 7.37 | ≥1.6 µg/mL |

*The LC-MS/MS value is greater-than 1.0 µg/mL, however for the LFA ≥1.0 µg/mL is interpreted as concentrations between 1.0 to <1.6 µg/mL. Discrepancies between LC-MS/MS and LFA-determined DTG concentrations are highlighted, all except sample ID 82 are within a difference of 0.1 µg/mL. Abbreviations: DTG, dolutegravir.

Figure S4. Flow diagram of the number of Eswatini samples with DTG detectable at or above daily trough concentrations (≥1 µg/mL) by LFA relative to the quantitated viral load. All samples were selected for resistance genotyping and therefore had VLs of at least 200 copies/mL (<1000 = 200–999 cp/mL). Of samples with VL ≥1000 cp/mL, only seven (7%) had DTG ≥1 µg/mL. These may be highest priority for drug resistance genotyping and focusing on this group could potentially reduce healthcare costs by avoiding genotyping individuals who are viremic because of poor treatment adherence. Abbreviations: DTG, dolutegravir; LFA, lateral flow assay; VL, viral load.
