# Supplementary material for: A Highly Sensitive and Specific Rapid Lateral Flow Assay for the HIV Integrase Strand Transfer Inhibitors Bictegravir, Cabotegravir, and Dolutegravir
Source: AIDS Res Treat. 2026 Oct 25;2026:4809477. doi: 10.1155/arat/4809477 (PMC13695850; doi:10.1155/arat/4809477)
Supplement: Supplementary file 1 [file ARAT-2026-4809477-s001.zip › Table S1.pdf]

Sample #	LC-MS/MS DTG (µg/mL)	LFA DTG Result
12	0.00	<1.0 µg/mL
13	0.00	<1.0 µg/mL
14	0.00	<1.0 µg/mL
17	0.00	<1.0 µg/mL
18	0.00	<1.0 µg/mL
21	0.00	<1.0 µg/mL
22	0.00	<1.0 µg/mL
23	0.00	<1.0 µg/mL
24	0.00	<1.0 µg/mL
27	0.00	<1.0 µg/mL
30	0.00	<1.0 µg/mL
38	0.00	<1.0 µg/mL
42	0.00	<1.0 µg/mL
43	0.00	<1.0 µg/mL
47	0.00	<1.0 µg/mL
49	0.00	<1.0 µg/mL
52	0.00	<1.0 µg/mL
53	0.00	<1.0 µg/mL
55	0.00	<1.0 µg/mL
56	0.00	<1.0 µg/mL
57	0.00	<1.0 µg/mL
63	0.00	<1.0 µg/mL
67	0.00	<1.0 µg/mL
68	0.00	<1.0 µg/mL
70	0.00	<1.0 µg/mL
73	0.00	<1.0 µg/mL
74	0.00	<1.0 µg/mL
81	0.00	<1.0 µg/mL
96	0.00	<1.0 µg/mL
99	0.00	<1.0 µg/mL
4	0.01	<1.0 µg/mL
8	0.01	<1.0 µg/mL
16	0.01	<1.0 µg/mL
20	0.01	<1.0 µg/mL

29	0.01	<1.0 μg/mL
33	0.01	<1.0 μg/mL
36	0.01	<1.0 μg/mL
45	0.01	<1.0 μg/mL
77	0.01	<1.0 μg/mL
88	0.01	<1.0 μg/mL
90	0.01	<1.0 μg/mL
92	0.01	<1.0 μg/mL
75	0.06	<1.0 μg/mL
100	0.06	<1.0 μg/mL
62	0.09	<1.0 μg/mL
94	0.10	<1.0 μg/mL
1	0.14	<1.0 μg/mL
51	0.18	<1.0 μg/mL
72	0.40	<1.0 μg/mL
66	0.44	<1.0 μg/mL
48	0.55	<1.0 μg/mL
41	0.61	<1.0 μg/mL
54	0.68	<1.0 μg/mL
59	0.77	<1.0 μg/mL
85	0.85	<1.0 μg/mL
79	0.90	<1.0 μg/mL
46	1.06	<1.0 μg/mL
82	1.20	≥1.6 μg/mL
39	1.21	≥1.0 μg/mL
71	1.29	≥1.0 μg/mL
98	1.53	≥1.0 μg/mL
40	1.58	≥1.0 μg/mL
2	1.60	≥1.0 μg/mL*
37	1.64	≥1.0 μg/mL*
80	1.67	≥1.0 μg/mL*
69	1.69	≥1.6 μg/mL
95	1.69	≥1.6 μg/mL
28	1.77	≥1.6 μg/mL
26	1.78	≥1.6 μg/mL
84	2.00	≥1.6 μg/mL
60	2.21	≥1.6 μg/mL

11	2.34	≥1.6 μg/mL
50	2.37	≥1.6 μg/mL
65	2.40	≥1.6 μg/mL
64	2.47	≥1.6 μg/mL
35	2.50	≥1.6 μg/mL
93	2.53	≥1.6 μg/mL
34	2.56	≥1.6 μg/mL
9	2.60	≥1.6 μg/mL
15	2.60	≥1.6 μg/mL
10	2.69	≥1.6 μg/mL
19	2.70	≥1.6 μg/mL
86	2.70	≥1.6 μg/mL
89	2.86	≥1.6 μg/mL
78	2.92	≥1.6 μg/mL
44	3.14	≥1.6 μg/mL
7	3.23	≥1.6 μg/mL
32	3.39	≥1.6 μg/mL
25	3.41	≥1.6 μg/mL
97	3.54	≥1.6 μg/mL
91	3.69	≥1.6 μg/mL
31	3.98	≥1.6 μg/mL
58	4.36	≥1.6 μg/mL
83	4.45	≥1.6 μg/mL
87	5.04	≥1.6 μg/mL
3	5.66	≥1.6 μg/mL
61	5.78	≥1.6 μg/mL
5	6.06	≥1.6 μg/mL
76	6.07	≥1.6 μg/mL
6	7.37	≥1.6 μg/mL

*The LC-MS/MS value is greater-than 1.0 μg/mL; however, for the LFA, ≥1.0 μg/mL is interpreted as concentrations between 1.0 to <1.6 μg/mL. Discrepancies between LC-MS/MS and LFA-determined DTG concentrations are highlighted, all except sample ID 82 are within a difference of 0.1 μg/mL. Abbreviations: DTG, dolutegravir.
